# Cost‐effectiveness of osimertinib versus placebo in resected EGFR‐mutated non‐small cell lung cancer in China

**DOI:** 10.1002/cam4.4798

**Published:** 2022-06-11

**Authors:** Xiwen Zhou, Jianting Du, Guobing Xu, Chun Chen, Bin Zheng, Jiahe Chen

**Affiliations:** ^1^ College of Finance Fujian Jiangxia University Fuzhou China; ^2^ Department of Thoracic Surgery Fujian Medical University Union Hospital Fuzhou Fujian China; ^3^ Key Laboratory of Cardio‐Thoracic Surgery (Fujian Medical University) Fujian Province University Fuzhou Fujian China; ^4^ Department of Pharmaceutical and Health Economics, School of Pharmacy University of Southern California Los Angeles California USA

**Keywords:** ADAURA trail, adjuvant therapy, cost‐effectiveness, non‐small cell lung cancer, osimertinib

## Abstract

**Purpose:**

We aim to assess whether osimertinib postoperative adjuvant therapy, compared with placebo, is cost‐effective in China.

**Methods:**

We set up the Markov model that contains three health states over a 20‐year period. Data were collected from the ADAURA trial that included transition probabilities and safety data. Through the analysis of literature and local charges, we explore both the cost and utility values. Sensitivity analyses were employed using TreeAge Pro software to access model stability.

**Findings:**

Patients in the osimertinib group had 1.46 more Quality‐adjusted Life Years (8.45 QALYs vs 6.99 QALYs) than the placebo group at an incremental cost of $14098.51($39962.99 vs $25864.48). Compared with the placebo group, the treatment strategy with osimertinib postoperative adjuvant therapy had an incremental cost‐effectiveness ratio of $9661.97/QALY. The probability of the osimertinib‐assisted therapy strategy being cost‐effective will reach 100% if the threshold of willingness to pay is above $15,000/QALY.

**Implications:**

From the perspective of the Chinese Healthcare System, the treatment strategy with osimertinib postoperative adjuvant therapy is more cost‐effective than the placebo strategy.

## Graphical abstract

The study founds that the treatment strategy with osimertinib postoperative adjuvant therapy is more cost‐effective than the placebo strategy through setting up a Markov model. The probability of osimertinib‐assisted therapy strategy being cost‐effective will reach 100% if the threshold of willingness to pay is above $15000/QALY in China.

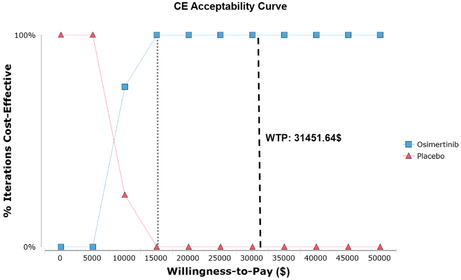

## 1 INTRODUCTION

Lung cancer remains the most common and deadliest malignancy throughout the world.^1^ Non‐small‐cell lung cancer (NSCLC) is the leading subtype and accounts for more than 85% of lung cancer.^2^ Approximately 30% of NSCLC patients present with surgically resectable tumors.^3^, ^4^, ^5^ Postoperative supplementary chemotherapy is suggested for complete resection of stage II to IIIA patients and subject to postoperative evaluation to assess the benefits and risks of selected stage IB patients.^6^ However, this treatment has only reduced a 16% risk of relapse or death of the disease and improved 5% of 5‐year overall survival rates.^7^, ^8^ Over a median of approximately 5 years of follow‐up time, the percentage of patients who have disease recurrence or who die after surgery remains high (ranging from 45% among patients with stage IB disease to 76% among those with stage IIIA disease), regardless of the use of postoperative chemotherapy.^8^

The epidermal growth factor receptor (EGFR) gene is one of the most frequent oncogenic‐driven mutations in NSCLC, where Ex19del and L858R are common mutations. For advanced NSCLC with EGFR mutation positive, EGFR tyrosine kinase inhibitors (EGFR‐TKIs) as first‐line treatment are recommended by oncologists.^9^, ^10^, ^11^, ^12^, ^13^, ^14^ The efficacy of EGFR‐TKIs in patients with advanced disease led to the investigation of their use as an adjuvant treatment for resectable disease. Studies have shown that disease‐free survival (DFS) may be longer among patients with resected NSCLC with EGFR mutation who receive adjuvant first‐generation EGFR‐TKIs than among those who receive adjuvant chemotherapy or placebo.^15^, ^16^

The phase III, randomized ADAURA trial assessed the efficacy and safety of osimertinib as supplementary treatment compared with placebo in patients with completely resected stage IB to IIIA NSCLC with EGFR mutant, after adjuvant chemotherapy, according to physician and patient choices. The study showed that the median follow‐up for DFS was significantly longer with osimertinib than with placebo (22.1 months vs. 14.9 months).^6^

For clinicians and decision makers, the more excellent cost‐effectiveness is a major factor, which may affect the choice of TKIs ultimately. As far as we know, there was no economic analysis evaluated osimertinib for adjuvant therapy in resected NSCLC. With a view of providing evidence for clinical and reimbursement decision‐making, the aim of the study was to assess the cost‐effectiveness of osimertinib in postoperative patients with completely resected IB to IIIA, EGFR‐mutated NSCLC. These patients can choose chemotherapy or no chemotherapy after surgery according to specific circumstances. In this study, a Markov decision model was constructed with real‐world data.

## 2 METHODS

To compare osimertinib and placebo for postoperative supplementary therapy in NSCLC, we conducted a cost‐effectiveness analysis utilizing a Markov model. In the research, we aimed to explore the benefit level from the Chinese health insurance, and hence, only direct medical costs were considered. The time horizon was 20 years. And, 5% annual discount rate was employed for costs.^17^

### 2.1 Study population

In this study, we used data from the ADAURA trial, one recently published article of phase III randomized controlled study (RCT). Patients were graded on the basis of tumor stage (IB, II, or IIIA), status of EGFR mutations, and patients’ race and were randomized in a 1:1 ratio to obtain placebo or osimertinib 80 mg once daily.^6^ Patients were screened and randomized after complete resection and chemotherapy, where complete removal of tumor is necessary. In the RCT ADAURA, all patients had been followed up for at least 12 months. The median duration of osimertinib treatment was 22.5 months (range, 0–38) and that of the placebo group was 18.7 months (range, 0–36). The population of patients who discontinued osimertinib or placebo was 92 (27%) and 174 (51%), respectively.^6^

### 2.2 Study model

**FIGURE 1:**
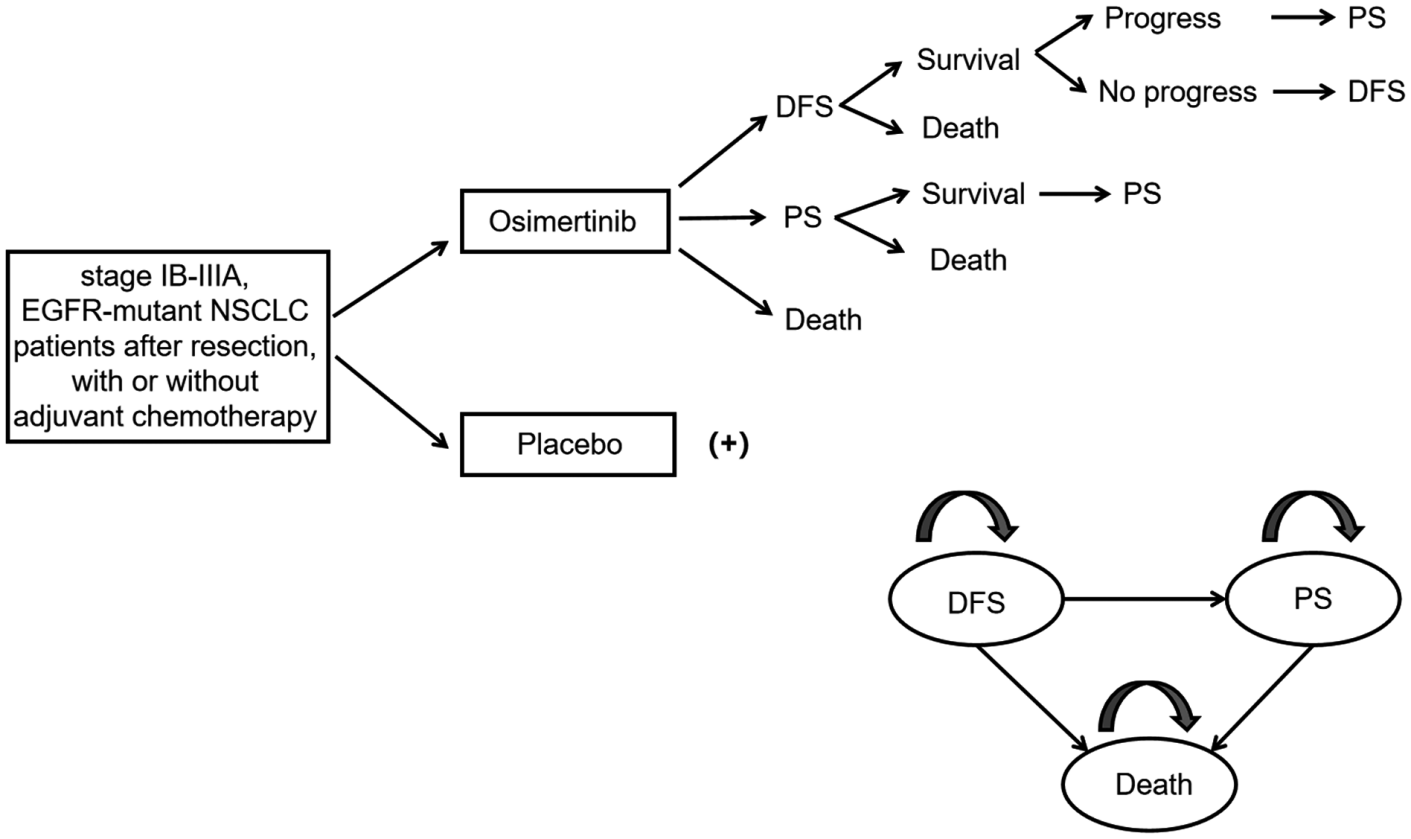
Schematic diagram of Markov model; DFS, disease‐free survival; PS, progressed survival

We established a Markov model based on the Chinese Healthcare System to analyze the cost‐effectiveness of osimertinib and placebo for supplementary therapy of completely resectable NSCLC patients with EGFR mutant using TreeAge Pro 2020 (TreeAge Softage, Williamstown, MA). All the cost data were collected from public literature and local charge. All costs were presented as the 2021 price employing the local Consumer Price Index for adjustment and expressed in 2021 US dollars (using a ratio of 1 US dollar = 6.5 Chinese Yuan).^18^ The overall mortality in any state was estimated as the combination between mortality due to lung cancer and background death rate for other reasons. The probability of natural death in DFS was calculated based on the 2010 census data of China.^19^ The DFS curve of the ADAURA trial and the overall survival(OS) curve of the FLAURA trial were extracted to estimate the probability of progressive death due to lung cancer using the GetData Graph Digitizer software Package (Version 2.26, getdata‐graph‐digitizer.com) (Table 1).^6^, ^20^, ^21^, ^22^ There are two branches at the decision node in the model, representing the osimertinib arm and placebo arm, respectively. The model consisted of three different health states, consisting of DFS, progressed survival (PS) as well as death, and was further ascertained by oncology specialists (Figure 1). All patients started in a DFS state. According to the clinicians’ experiences, most patients with early resected NSCLC were over 60‐year old, so it is assumed that all patients died after 20 years in the model. The period was set to 1 month in the Markov model to facilitate the calculation of parameters, and the simulation period was set to 20 years to reflect the disease procession. During each simulation cycle, patients could remain stable in DFS, have progression, or die. Patients in DFS take osimertinib for 3 years at most.^6^ When patients proceed to the progressive disease state, they either remained or transitioned to death. In the ADAURA study, researchers suggested post‐line treatment after progression was initiated at the clinicians' discretion. Therefore, the data of post‐line treatment were hypothesized to be the same in both groups. We modeled the disease progression until all patients arrived in the death state, namely the final state. Data of survival and DFS were collected from the published article of the ADAURA trial, and cost and utility data were based on literature and local charge. The major outcomes in the model were the cost‐effectiveness of two strategies, which were measured by the incremental cost per quality‐adjusted life years (QALYs). QALYs were calculated by combing survival time and health‐related quality of life (HRQOL) (a health state value from 0 for death to 1 for perfect health). We speculated on both strategies’ cost‐effectiveness by estimating the incremental cost‐effectiveness ratio (ICER). The ICER was defined as the ratio of each strategy incremental cost to its incremental effectiveness. The World Health Organization (WHO) recommended that the intervention should be considered cost‐effective if the ICER is between 1 and 3 times the gross domestic product (GDP) per capita, and the strategy with ICER values higher than 3 times the GDP per capita may be considered not worthwhile.^23^ Therefore, we used 3 times the GDP per capita in the People's Republic of China in 2020($31451.64) as the cost‐effectiveness threshold in Chinese contexts,^17^, ^24^, ^25^ which means the ICER value of the osimertinib treatment group should be less than $31451.64/QALY compared to that of the placebo group, accord with the Chinese social preference.

**TABLE 1:** Key model input parameters

Input Parameter	Value	Source (Reference)
Weibull DFS survival model of Osimertinib Stage **I**B**–III**A population	**λ =** 0.000112076; **γ =** 2.162643	6
Gompertz DFS survival model of Placebo Stage **I**B**–III**A population	**λ =** 0.0131044; **γ =** 0.425736	6
Probability of SAEs in osimertinib therapy	0.23 (0.1725–0.2875)	21, 22

Abbreviations: DFS, disease‐free survival; SAEs, severe adverse effects.

### 2.3 Model inputs

Only direct healthy costs were analyzed in our model, consisting of the costs of drug acquisition and adverse event management. Study‐related treatment costs applied to the analysis are listed in Table 2.^21^, ^26^, ^27^, ^28^, ^29^ The costs of all strategies were calculated on the basis of costs per month (30 days). We used the current retail price in 2021 from the National Healthcare Security Administration to estimate the cost of osimertinib. The costs of medical services and drug administration were from the National Healthcare Security Administration base rate in 2021. All costs of adverse event management were median drug prices according to relevant literature and local charges.^21^ Because we hypothesized that palliative treatment costs after disease progression and this terminal care after 12‐month health‐care following progression were not different between the two arms, we excluded them from the analysis. Efficacy data measured as DFS of osimertinib and placebo were derived from the ADAURA trial. Cox proportional hazard ratio (HR) was estimated for DFS (HR = 0.20, 99.12% CI, 0.14–0.30, *p* < 0.001).^6^ Serious adverse effects (SAE) were defined as treatment‐associated ≥ grade 3 events. We obtained adverse event probabilities for both treatments from relevant literature.^20^, ^21^

**TABLE 2:** Base‐case cost estimates and utilities

Input Parameter	Value (Range)	Source (Reference)
Cost of osimertinib per cycle, US $	858.46 (429.23–858.46)	Local charge
Cost of follow‐up per cycle, US $	60.3 (45.5–75.1)	Local charge
Cost of SAEs per unit, US $	362 (272–453)	22
Cost of supportive care per cycle, US $	359 (169–845)	22
Utility in DFS	0.82 (0.78–0.86)	24,26,27
Utility in PS	0.70 (0.66–0.74)	28
Disutility of SAE	−0.0731 (−0.0731–0)	28

Abbreviations: DFS, disease‐free survival; PS, progressed survival; SAEs = severe adverse effects.

### 2.4 Sensitivity analysis

To explore how results varied across plausible ranges, we performed a series of sensitivity analyses. All variables in the model were varied across a reasonable range, which were required from certainty intervals (CIs). We do a one‐way sensitivity analysis on price, as in current China, the price will not go higher. Uncertainty in all model parameters was investigated simultaneously by running a Monte Carlo simulation with 1000 iterations. We assessed the 20‐year cost and effectiveness of different treatment regimens and employed 95% certainty intervals to express the uncertainty around these estimates. The ICERs from simulations were then compared with the willingness‐to‐pay (WTP) threshold value.

## 3 RESULTS

### 3.1 Base case analysis

**FIGURE 2:**
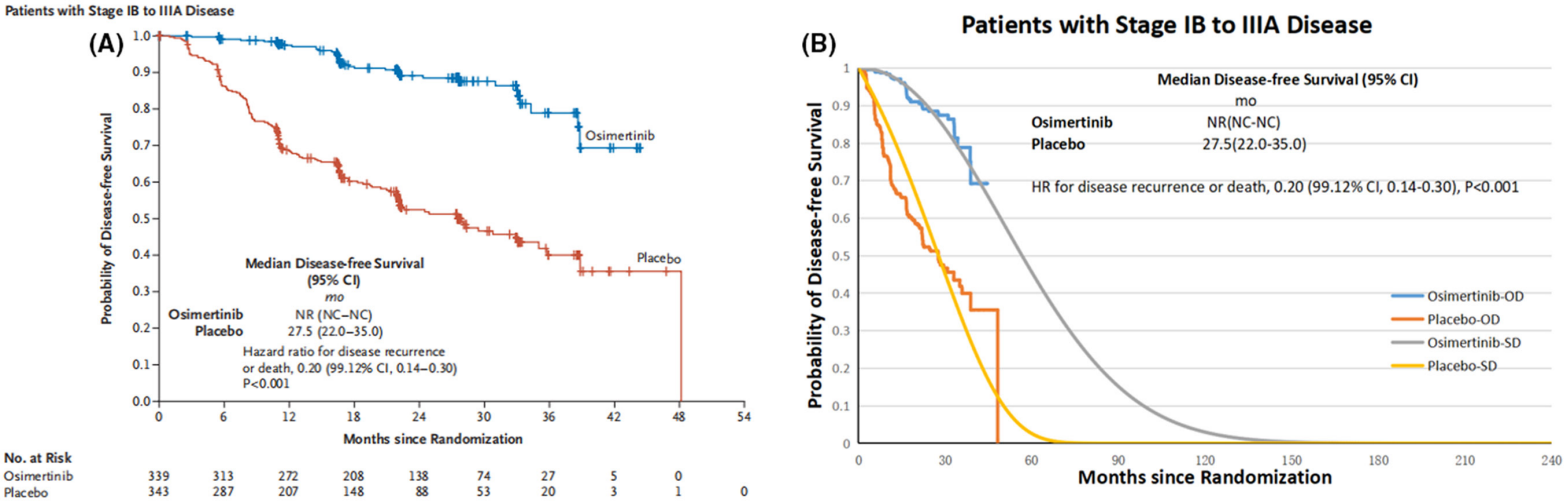
(A) Kaplan–Meier curve of progression‐free survival from the ADAURA trial^6^; (B) Fitted Kaplan–Meier models with Weibull tails for DFS; OD, original data; SD, simulation data

Figure 2 shows the results of the survival curve simulation. We found that the fitting data were well fitted with the original data, and the similarity could be observed from the fitted DFS and OS curves. We can take into account that the fitted data are rational and acceptable. Patients in the osimertinib group had 1.46 more QALYs (8.45 QALYs vs. 6.99 QALYs) compared to the placebo group at an incremental cost of $14098.51($39962.99 vs. $25864.48). Compared with the placebo group, the treatment strategy with osimertinib postoperative adjuvant therapy had an ICER of $9661.97/QALY, as shown in Table 3. It was estimated that the strategy with osimertinib postoperative adjuvant therapy was more efficient (1.46 QALYs) but more expensive than the placebo group ($14098.51).

**TABLE 3:** Summary of cost and outcome results from a base‐case analysis

Strategy	Costs, $	ΔCosts, $	QALYs, $	ΔQALYs, $	ICER, $/QALY
Placebo	25864.48	/	6.99	/	/
Osimertinib	39962.99	14098.51^a^	8.45	1.46^a^	9661.97^a^

Abbreviations: ICER, incremental cost‐effectiveness ratio; QALY, quality‐adjusted life year; /, No comparison.

^a^ Compared with placebo care after complete resection of the primary NSCLC.

### 3.2 Sensitivity analysis

**FIGURE 3:**
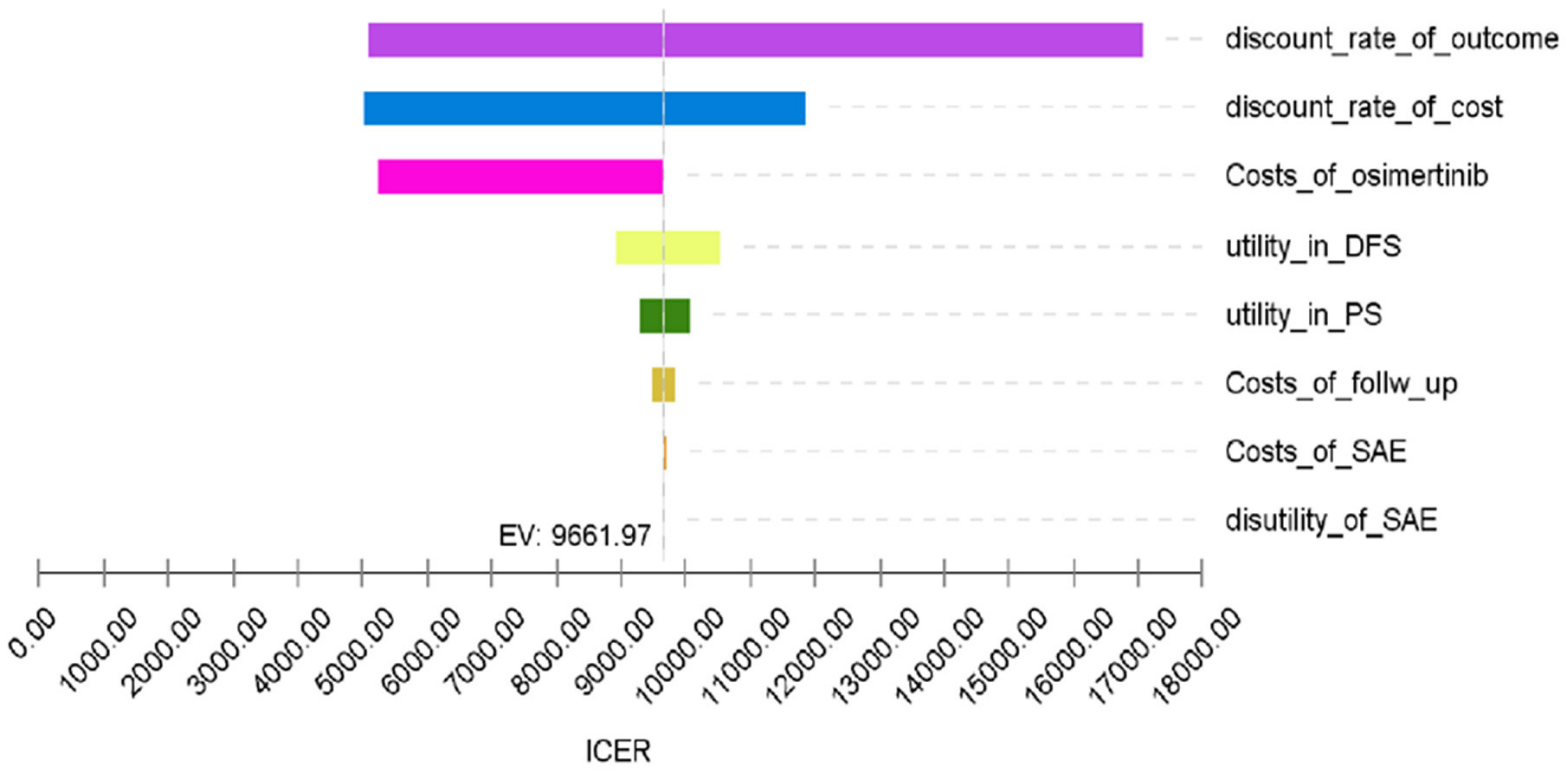
Tornado diagram of osimertinib cost‐effectiveness in China; DFS, disease‐free survival; PS, progressed survival; SAE, severe adverse effect

To examine how responsive the model and robust the base‐case analysis is, a one‐way sensitivity analysis was implemented between the osimertinib postoperative supplementary treatment group and the placebo strategy group. The results are summarized in a tornado diagram (Figure 3). Figure 3 shows that the discount rate of outcome indicated the most predominant effect on the results. Other variables, such as the discount rate of cost, costs of osimertinib, utility in DFS, utility in PS, costs of follow‐up, costs of SAE, and disutility of SAE, had a secondary or minor effect on the outcomes of the economy.

**FIGURE 4:**
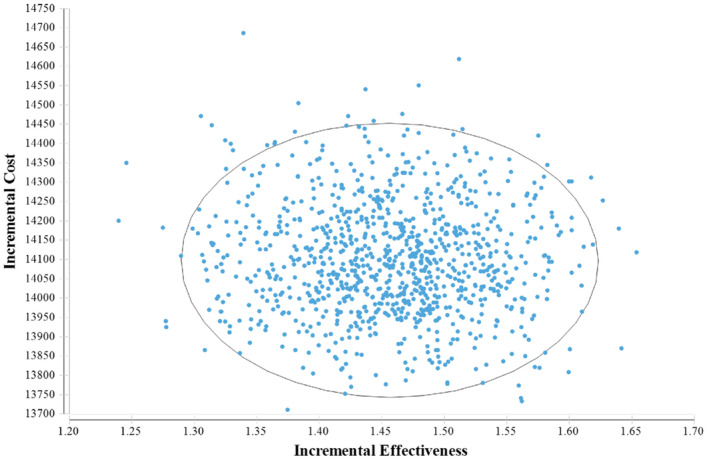
A probabilistic sensitivity analysis of osimertinib postoperative adjuvant therapy; each dot represents the ICER for one simulation. An ellipse means a 95% confidence interval

**FIGURE 5:**
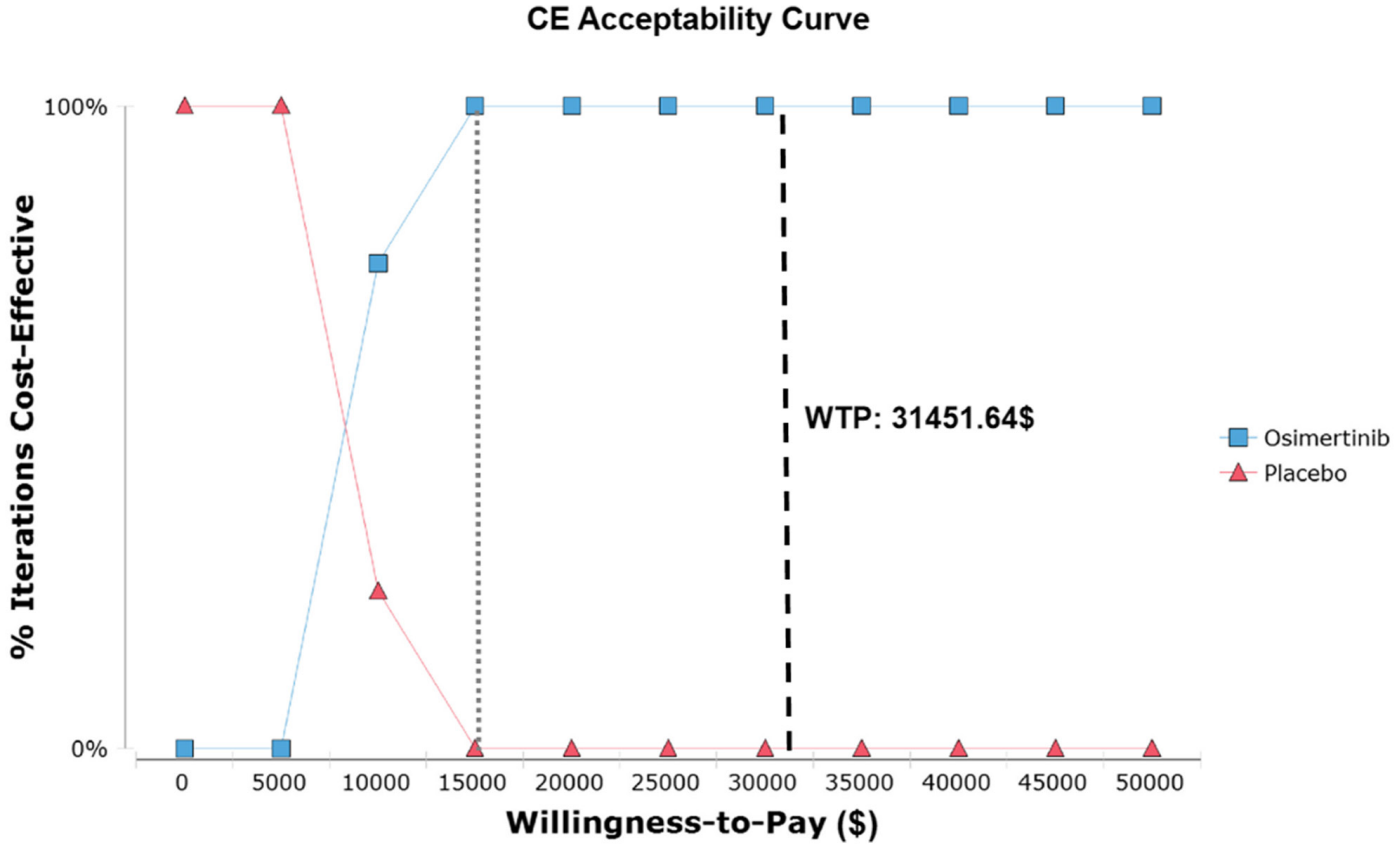
Cost‐effectiveness acceptability curve. CE, cost‐effectiveness; WTP, willing to pay

A probabilistic sensitivity analysis was conducted using 1000 Monte Carlo simulation cases to explore the effects of all input parameters on model outputs, as shown in a scatter plot (Figure 4). The scatter plot includes an ellipse and an undisplayed threshold line. The WTP threshold ($31451.64) is much greater than the ICER value, so the line is not shown in the graph. The scatter diagram further demonstrated that the ICER of 1000 Monte Carlo simulations is focused on the effective dominant quadrant and far below the WTP threshold. For probabilistic sensitivity analysis, cost‐effectiveness acceptability curves (Figure 5) indicate that the osimertinib postoperative adjuvant therapy strategy is more cost‐effective than the placebo strategy as a WTP threshold increased. The probability of the osimertinib strategy being cost‐effective will reach 100% if the WTP threshold is above $15,000/QALY.

## 4 DISCUSSION

As far as we know that the study is a new finding that has not been reported in the previous literature. Our main purpose of building mathematical models is to investigate the cost‐effectiveness comparing both strategies, in patients who were diagnosed with stage IB to IIIA EGFR mutation NSCLC. These results preliminarily indicated that the incremental effectiveness and ICER of osimertinib postoperative adjuvant therapy compared with the placebo strategy were 1.46 QALYs and $9661.97/QALY, respectively. The ICER of the osimertinib postoperative adjuvant therapy compared with the placebo strategy was far less than the threshold (WTP = $31451.64/QALY) by incremental cost‐effectiveness analysis.

However, our study still exists several limitations. First, there are some uncertainties at each stage of the model. However, we performed a series of sensitivity analyses to stably support the results of the analysis. Second, there is no accepted cost‐effectiveness threshold for Chinese national conditions now. According to the suggestion in the “China Guidelines for Pharmacoeconomic Evaluations,”^17^ triple GDP per capita in China is served as the threshold, which is recommended by the WHO and also widely accepted by Chinese investigators.^30^ Third, owing to the absence of local health data, the utility scores of this study were only available by reference to literature published abroad when the QALYs were compared between groups.^25^, ^26^, ^27^, ^28^, ^29^ Whereas, different ethnocultural backgrounds lead to the different situations for each state. Thus, this study simulates the outcomes of disease, without considering the utility of the drug reaction.^30^ Fourth, our study was based on individual patient data from the ADAURA trial. In the ADAURA trial, most of the patients in both groups were still alive at 24 months, and the OS estimate is still immature.^6^ Thus, we evaluated the probability of patients in each state by combining the natural Chinese population mortality^18^ and the OS data of the FLAURA trial^19^ when we established the model. Fifth, this study did not consider the impact of grades 2 or below adverse events on the results, which may result in the underestimation of the effectiveness of osimertinib.^21^ Nonetheless, this does not affect our positive conclusion of this study. Finally, we assumed that all patients in the study continue treatment with osimertinib after disease progression and ignored the importance of individualized therapy. For instance, patients with advanced NSCLC may have different treatment regimens but did not discuss the differences of various strategies in this study. However, due to the recent changes in the prices of osimertinib, increasing clinical experts suggested using osimertinib to continue treatment after disease progression.

## 5 CONCLUSION

According to our analysis, from a health‐care system perspective, the osimertinib postoperative adjuvant treatment strategy has been economical and effective for NSCLC patients with resectable EGFR mutation in China. These findings may have more general implications for guiding therapeutic decisions and health‐care requests in EGFR‐mutated NSCLC. And, we believed that the findings could be easily popularized to other countries and regions with a better health‐care system.

## CONFLICT OF INTEREST

All authors have no relevant financial or other relationships to disclose.

## AUTHOR CONTRIBUTIONS

C JH conceived and designed the study. D JT and Z XW interpretation of data and drafted the manuscript. CC and X GB the acquisition and analysis. ZB reviewed and edited the manuscript. All authors read and approved the final manuscript.

## ETHICS STATEMENT

The study was exempt from gaining individual consent, and no ethical approval was required for the study.

## Data Availability

The datasets used and analyzed during the current study are available from the corresponding author on reasonable request.

## References

[1] Erratum to "Cancer statistics, 2021". CA Cancer J Clin 2021;71(4):359. 10.3322/caac.21669 34232515

[2] American Cancer Society . Lung cancer (non‐small cell). Available form: http://www.cancer.org/cancer/lung‐cancer.html. Accessed April 5, 2020.

[3] Datta D , Lahiri B . Preoperative evaluation of patients undergoing lung resection surgery. Chest. 2003;123(6):2096‐2103. doi:10.1378/chest.123.6.2096 12796194

[4] Cagle PT , Allen TC , Olsen RJ . Lung cancer biomarkers: present status and future developments. Arch Pathol Lab Med. 2013;137(9):1191‐1198. doi:10.5858/arpa.2013-0319-CR 23991729

[5] Le Chevalier T . Adjuvant chemotherapy for resectable non‐small‐cell lung cancer: where is it going? Ann Oncol. 2010;21(Suppl 7):vii196‐vii198. doi:10.1093/annonc/mdq376 20943614

[6] Wu YL , Tsuboi M , He J , et al. Osimertinib in resected EGFR‐mutated non‐small‐cell lung cancer. N Engl J Med. 2020;383(18):1711‐1723. doi:10.1056/NEJMoa2027071 32955177

[7] Kris MG , Gaspar LE , Chaft JE , et al. Adjuvant systemic therapy and adjuvant radiation therapy for stage I to IIIA completely resected non‐small‐cell lung cancers: American society of clinical oncology/cancer care Ontario clinical practice guideline update. J Clin Oncol. 2017;35(25):2960‐2974. doi:10.1200/JCO.2017.72.4401 28437162

[8] Pignon JP , Tribodet H , Scagliotti GV , et al. Lung adjuvant cisplatin evaluation: a pooled analysis by the LACE Collaborative Group. J Clin Oncol. 2008;26(21):3552‐3559. doi:10.1200/JCO.2007.13.9030 18506026

[9] Hanna N , Johnson D , Temin S , et al. Systemic therapy for stage IV non‐small‐cell Lung cancer: American society of clinical oncology clinical practice guideline update. J Clin Oncol. 2017;35(30):3484‐3515. doi:10.1200/JCO.2017.74.6065 28806116

[10] Planchard D , Popat S , Kerr K , et al. Metastatic non‐small cell lung cancer: ESMO Clinical Practice Guidelines for diagnosis, treatment and follow‐up. Ann Oncol. 2018;29(Suppl 4):iv192‐iv237. doi:10.1093/annonc/mdy275 30285222

[11] Wu YL , Planchard D , Lu S , et al. Pan‐Asian adapted Clinical Practice Guidelines for the management of patients with metastatic non‐small‐cell lung cancer: a CSCO‐ESMO initiative endorsed by JSMO, KSMO, MOS. SSO and TOS Ann Oncol. 2019;30(2):171‐210. doi:10.1093/annonc/mdy554 30596843

[12] Rosell R , Carcereny E , Gervais R , et al. Erlotinib versus standard chemotherapy as first‐line treatment for European patients with advanced EGFR mutation‐positive non‐small‐cell lung cancer (EURTAC): a multicentre, open‐label, randomised phase 3 trial. Lancet Oncol. 2012;13(3):239‐246. doi:10.1016/S1470-2045(11)70393-X 22285168

[13] Mok TS , Wu YL , Thongprasert S , et al. Gefitinib or carboplatin‐paclitaxel in pulmonary adenocarcinoma. N Engl J Med. 2009;361(10):947‐957. doi:10.1056/NEJMoa0810699 19692680

[14] Sequist LV , Yang JC , Yamamoto N , et al. Phase III study of afatinib or cisplatin plus pemetrexed in patients with metastatic lung adenocarcinoma with EGFR mutations. J Clin Oncol. 2013;31(27):3327‐3334. doi:10.1200/JCO.2012.44.2806 23816960

[15] Cheng H , Li XJ , Wang XJ , et al. A meta‐analysis of adjuvant EGFR‐TKIs for patients with resected non‐small cell lung cancer. Lung Cancer. 2019;137:7‐13. doi:10.1016/j.lungcan.2019.08.002 31520922

[16] Huang Q , Li J , Sun Y , Wang R , Cheng X , Chen H . Efficacy of EGFR tyrosine kinase inhibitors in the adjuvant treatment for operable non‐small cell lung cancer by a meta‐analysis. Chest. 2016;149(6):1384‐1392. doi:10.1016/j.chest.2015.12.017 26836897

[17] Liu G . 2020 China Guidelines for Pharmacoeconomic Evaluations and Manual. Science Press; 2020.

[18] Monetary Policy Department of The People's Bank of China . Historical data of the central parity of RMB exchange rate. Available from: http://www.pbc.gov.cn/zhengcehuobisi/125207/125217/125925/index.html. Accessed April 21, 2021.

[19] The Census Office of the State Council and the Department of Population and Employment Statistics of the National Bureau of Statistics, China 2010 Census Data. 2012: China Statistics Publishing House.

[20] Soria JC , Ohe Y , Vansteenkiste J , et al. Osimertinib in untreated EGFR‐mutated advanced non‐small‐cell lung cancer. N Engl J Med. 2018;378(2):113‐125. doi:10.1056/NEJMoa1713137 29151359

[21] Mok TS , Wu Y‐L , Ahn M‐J , et al. Osimertinib or platinum‐pemetrexed in EGFR T790M‐positive lung cancer. N Engl J Med. 2017;376(7):629‐640. doi:10.1056/NEJMoa1612674 27959700 PMC6762027

[22] Wu B , Gu X , Zhang Q . Cost‐effectiveness of Osimertinib for EGFR mutation‐positive non‐small cell lung cancer after progression following first‐line EGFR TKI therapy. J Thorac Oncol. 2018;13(2):184‐193. doi:10.1016/j.jtho.2017.10.012 29101057

[23] Bertranou E , Bodnar C , Dansk V , Greystoke A , Large S , Dyer M . Cost‐effectiveness of osimertinib in the UK for advanced EGFR‐T790M non‐small cell lung cancer. J Med Econ. 2018;21(2):113‐121. doi:10.1080/13696998.2017.1377718 28880737

[24] International Monetary Fund, World Economic Outlook database: April 2021.

[25] International Society for Pharmacoeconomics and Outcomes Research (ISPOR) . Pharmacoeconomic guidelines around the world. Available from: https://tools.ispor.org/peguidelines/. Accessed June 19, 2020.

[26] Lu S , Ye M , Ding L , Tan F , Fu J , Wu B . Cost‐effectiveness of gefitinib, icotinib, and pemetrexed‐based chemotherapy as first‐line treatments for advanced non‐small cell lung cancer in China. Oncotarget. 2017;8(6):9996‐10006. doi:10.18632/oncotarget.14310 28036283 PMC5354787

[27] Grutters JP , Joore MA , Wiegman EM , et al. Health‐related quality of life in patients surviving non‐small cell lung cancer. Thorax. 2010;65(10):903‐907. doi:10.1136/thx.2010.136390 20861294

[28] Chouaid C , Agulnik J , Goker E , et al. Health‐related quality of life and utility in patients with advanced non‐small‐cell lung cancer: a prospective cross‐sectional patient survey in a real‐world setting. J Thorac Oncol. 2013;8(8):997‐1003. doi:10.1097/JTO.0b013e318299243b 23787802

[29] Li WQ , Li LY , Chai J , Cui JW . Cost‐effectiveness analysis of first‐line treatments for advanced epidermal growth factor receptor‐mutant non‐small cell lung cancer patients. Cancer Med. 2021;10(6):1964‐1974. doi:10.1002/cam4.3733 33626238 PMC7957173

[30] Cai H , Zhang L , Li N , et al. Cost‐effectiveness of Osimertinib as first‐line treatment and sequential therapy for EGFR mutation‐positive non‐small cell lung cancer in China. Clin Ther. 2019;41(2):280‐290. doi:10.1016/j.clinthera.2018.12.007 30639208

